# Towards equitable access to medicines for the rural poor: analyses of insurance claims reveal rural pharmacy initiative triggers price competition in Kyrgyzstan

**DOI:** 10.1186/1475-9276-8-43

**Published:** 2009-12-14

**Authors:** Brenda Waning, Jason Maddix, Yorghos Tripodis, Richard Laing, Hubert GM Leufkens, Manjusha Gokhale

**Affiliations:** 1 Boston University School of Medicine, Department of Family Medicine; One Boston Medical Center Place, Dowling 5 South, Boston, MA 02118, USA; 2 Boston University School of Public Health, Department of Biostatistics Crosstown, 3rd floor, 801 Massachusetts Avenue, Boston, MA 02118 USA; 3 World Health Organization, Department of Policy, Standards, and Medicines CH-1211 Geneva 27, Switzerland; 4 Utrecht University, Division of Pharmacoepidemiology and Pharmacotherapy PO Box 80 082, 3508 TB, Utrecht, the Netherlands; 5 Boston University School of Public Health, Data Coordinating Center Crosstown, 3rd floor, 801 Massachusetts Avenue, Boston, MA 02118 USA

## Abstract

**Background:**

A rural pharmacy initiative (RPI) designed to increase access to medicines in rural Kyrgyzstan created a network of 12 pharmacies using a revolving drug fund mechanism in 12 villages where no pharmacies previously existed. The objective of this study was to determine if the establishment of the RPI resulted in the unforeseen benefit of triggering medicine price competition in pre-existing (non-RPI) private pharmacies located in the region.

**Methods:**

We conducted descriptive and multivariate analyses on medicine insurance claims data from Kyrgyzstan's Mandatory Health Insurance Fund for the Jumgal District of Naryn Province from October 2003 to December 2007. We compared average quarterly medicine prices in competitor pharmacies before and after the introduction of the rural pharmacy initiative in October 2004 to determine the RPI impact on price competition.

**Results:**

Descriptive analyses suggest competitors reacted to RPI prices for 21 of 30 (70%) medicines. Competitor medicine prices from the quarter before RPI introduction to the end of the study period decreased for 17 of 30 (57%) medicines, increased for 4 of 30 (13%) medicines, and remained unchanged for 9 of 30 (30%) medicines. Among the 9 competitor medicines with unchanged prices, five initially decreased in price but later reverted back to baseline prices. Multivariate analyses on 19 medicines that met sample size criteria confirm these findings. Fourteen of these 19 (74%) competitor medicines changed significantly in price from the quarter before RPI introduction to the quarter after RPI introduction, with 9 of 19 (47%) decreasing in price and 5 of 19 (26%) increasing in price.

**Conclusions:**

The RPI served as a market driver, spurring competition in medicine prices in competitor pharmacies, even when they were located in different villages. Initiatives designed to increase equitable access to medicines in rural regions of developing and transitional countries should consider the potential to leverage medicine price competition as a means of achieving their goal. Evaluations of interventions to increase rural access to medicines should include impact assessment on both formal and informal pharmaceutical markets.

## Background

Equitable access to medicines remains a challenge in developing and transitional countries, especially among the rural poor. Pharmacies in densely populated areas are always more lucrative, often leaving sparsely-populated rural regions without access to reliable sources of medicines within reasonable proximity. Even when pharmacies are physically present, medicines are often unaffordable, and their availability can be erratic because of failing public financing and supply chain management systems [1-11]. Understanding that a large number of people in developing countries seek care and medicines from the private sector, numerous private sector interventions have been mounted; however, a 2007 systematic review of private sector interventions on quality and utilization of care by the poor revealed an insufficient evidence base for those wishing to increase access to health services through private sector interventions [12].

One of the more commonly used mechanisms to address inequities in rural access to medicines has been the establishment of revolving drug funds, whereby a capital investment allows for the initial purchase of medicines and revenues from medicine sales or user fees are used to replenish stock. Sustainable and successful schemes have been described across Africa, South East Asia, and the Former Soviet Union [13-22]. More frequently, however, the literature reveals the failure of revolving drug funds to accomplish their objectives [14,15,22-34].

The design and management challenges of revolving drug funds that Cross *et al *[22] described in 1986 remain relevant today, nearly a quarter of a century later. Most noteworthy for our study is the inability of most schemes to adopt a business approach to their operations and practices, including a failure to assess the potential market and insufficient planning and marketing [22]. The concept of revolving drug funds has evolved into more sophisticated, business-focused initiatives, such as the Tanzanian Accredited Drug Dispensing Outlets and the Ghanaian CAREshops [6,35]. However, we have found no evidence that either these more advanced initiatives or the traditional revolving drug funds have been described or evaluated with regard to their impact on the existing pharmaceutical market in a given region.

Kyrgyzstan, like many developing and transitional countries, struggles to ensure access to medicines in rural regions. Approximately 64% of Kyrgyzstanis live in predominantly mountainous rural regions [36]. In participatory research sessions involving more than 80% of households in Naryn Province (n = 27,266), rural residents prioritized geographic access to pharmacies as the number one determinant of health in their communities [37]. In 2004, it was estimated that more than 300 rural villages in Kyrgyzstan had no physical access to pharmacies and medicines [38]. A number of factors underlie this absence of rural pharmacies: all pharmacies were privatized during health reforms following the dissolution of the Soviet Union, and would-be entrepreneurs believed pharmaceutical markets in rural regions were insufficient and unviable. A shortage of pharmacists in rural areas, combined with national policies that mandate pharmacies be staffed by pharmacists, created yet another deterrent to starting rural pharmacies.

When pharmacies are present in rural Kyrgyzstan, medicines are often unaffordable to the poor. The Kyrgyzstan Mandatory Health Insurance Fund covers medicines for approximately 80% of the population [39]. This insurance benefit, however, is administered through contracted private pharmacies concentrated in highly populated regions, and although rural residents are eligible for the medicines insurance benefit, they live too far away from contracted pharmacies to actually access it. Meanwhile, outpatient medicine purchases were the fastest growing component of out-of-pocket health expenditures from 2000 to 2003, increasing more than two-fold over this time period [40]. A 2005 evaluation in Jumgal District found that out-of-pocket costs for treatment of hypertension can represent up to 71% of non-food consumption per capita [38].

In 2005, the Kyrgyz Ministry of Health responded to the pharmacist human resource issue by changing the law to allow nurses to dispense medicines in pharmacies in rural regions after completing a two-week training course. A non-governmental organization (NGO), in collaboration with the Kyrgyz-Swiss Health Reform Support Project, Jumgal Village Health Committees, and the Kyrgyzstan Mandatory Health Insurance Fund, launched a rural pharmacy initiative (RPI) in Jumgal District. The RPI established pharmacies in 12 villages under a revolving drug fund mechanism. The RPI pharmacies were located in government-owned clinics and contracted with nurses already in the clinics to dispense medicines. To avoid disrupting the private market, the RPI management refrained from setting up pharmacies in Chaek, the district center, where a few pharmacies already existed. These private pharmacies also had outlets in two larger villages in Jumgal. A description of key features of the RPI is provided in Table 1.

**Table 1:** Key features of the Rural Pharmacy Initiative

Key Feature	Description
**Buy-in and Support**	Popular consensus that access to medicines is the number one health determinant in communities

	Involvement of Village Health Committees in the design of the RPI and refurbishment of pharmacy outlets

	Political will of the Kyrgyz authorities, and support from international organizations and the Mandatory Health Insurance Fund

**Cost Savings and Income**	Co-location of RPI outlets in existing government- owned health clinics, resulting in free rent and utilities

	Co-location of the RPI headquarters in government offices in the capital city, resulting in free rent and utilities

	Revenue stream assured by contractual arrangement between RPI pharmacies and the Mandatory Health Insurance Fund for administration of state-funded medicines benefit

**Human Resources and Oversight**	Contractual arrangements with existing nurses, paying them a modest bonus for their part-time pharmacy activities

	Availability of a highly-qualified pharmacist to manage the central RPI warehouse

	NGO managers' exceptional technical capacity in pharmaceutical management and their contributions of a great deal of personal time to support the RPI

While no distinct policy was created to establish medicine prices in the RPI, the management applied minimal mark-ups sufficient to cover their estimated operating costs. Retail mark-ups initially averaged approximately 30-50% for most medicines. Surprisingly, as the rural pharmacy initiative emerged, the private pharmacies in the district center appeared to be changing their prices on key medicines in order to compete with the new RPI pharmacies in area villages. Anecdotal reports and interviews with owners of private pharmacies in Chaek suggested that the RPI had an unplanned impact on overall medicine prices, even in the district center where the RPI was not operating.

The potential of these types of rural pharmacy initiatives to induce medicine price competition has profound implications for Kyrgyzstan and beyond. While scores of studies have been conducted to describe the unaffordability of medicines [7,9,11], few publications provide evidence-based guidance on how to decrease medicine prices so they are more affordable. The purpose of this study, therefore, is to determine if the RPI actually achieved the unforeseen benefit of triggering price competition in nearby private competitor pharmacies.

## Methods

**Figure 1:**
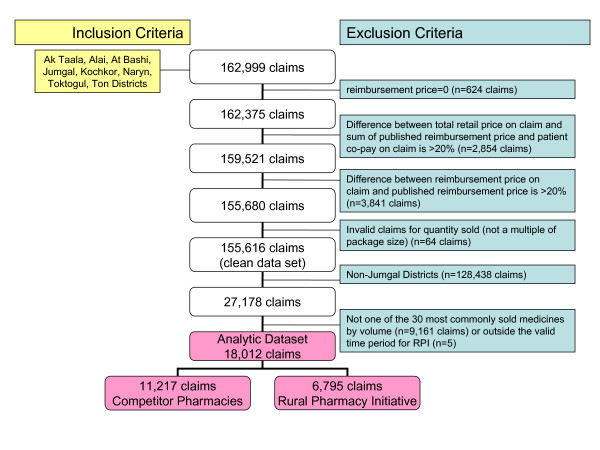
**Creation of Analytic Data Set from Medicines Insurance Claims**.

We obtained six lists of medicines covered by the Kyrgyz Mandatory Health Insurance Fund from 2002-2007 along with pharmaceutical claims data (n = 162,999 claims) for the period October 2003-December 2007 for the districts Ak Taala, Alai, At Bashi, Jumgal, Kochkor, Naryn, Toktogul, and Ton. We cleaned insurance claims data in several steps, excluding the following: reimbursement equaled zero; those where patient co-pay plus reimbursement did not equal total reimbursement; those with invalid package sizes; and those where the difference in published and actual reimbursement rates exceeded 20%. Differences in published and actual reimbursement prices result from a delay in actually distributing the revised published lists to the more than 300 contracted pharmacies throughout the region. We then excluded all non-Jumgal claims and all claims for medicines not on the list of top 30 selling medicines by volume, resulting in a final analytic data set of 18,012 Jumgal claims, which included 6,795 and 11,217 claims from RPI and competitor pharmacies, respectively (Figure 1).

We examined RPI and competitor prices using both simple descriptive and multivariate analyses. Since the RPI was first introduced in October 2004, this study period allows for a one-year observation period of competitor medicines prices before the introduction of the RPI and more than three years of observation for both RPI and competitor pharmacies afterwards. All prices are provided as price per unit (price per tablet or price per injection) in Kyrgyz Som.

**Figure 2:**
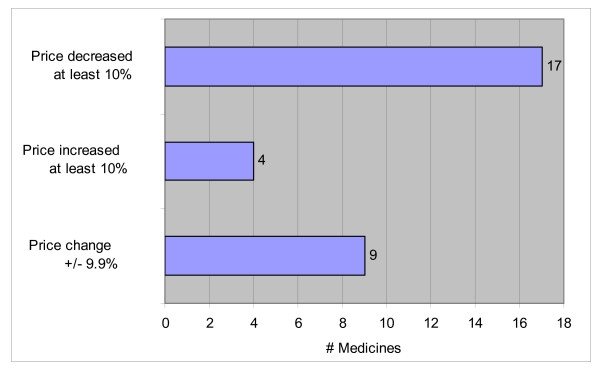
**Competitor medicine price changes after the RPI introduction**. Price change from quarter before observation of first RPI price to last study observation price.

**Figure 3:**
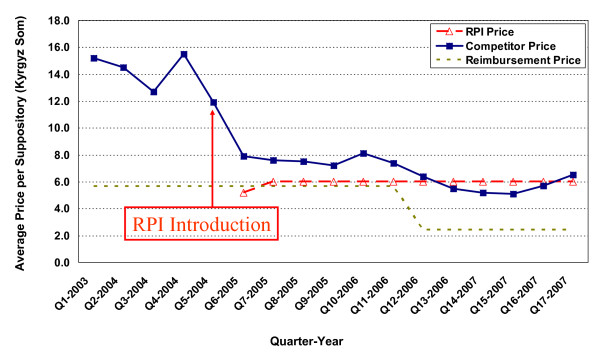
**Price changes for metronidazole 500 mg vaginal suppositories**.

**Figure 4:**
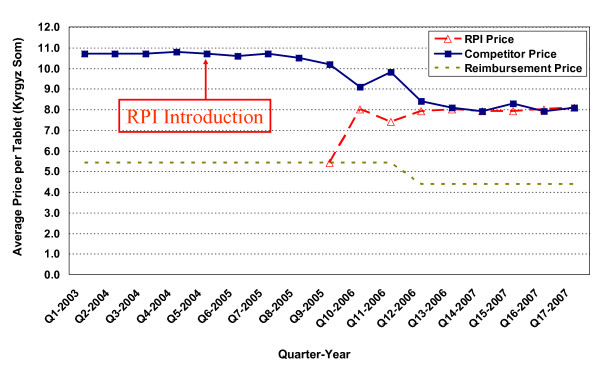
**Price changes for enalapril 20 mg tablets (Ednyt^®^)**.

**Figure 5:**
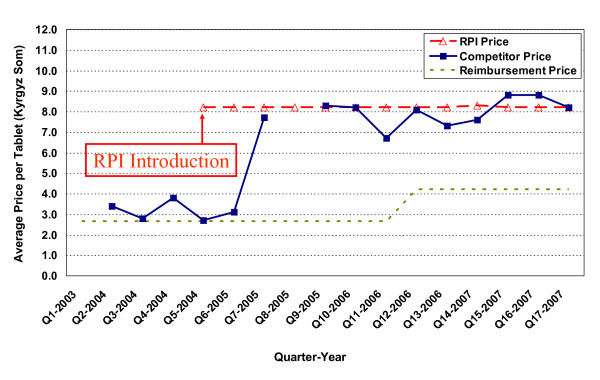
**Price changes for ferrous sulfate+ascorbic acid (Gyno-Tardyferon^®^) tablets**.

**Figure 6:**
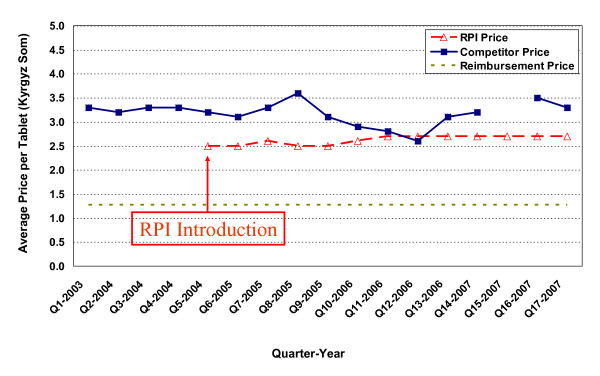
**Price changes for erythromycin 250 mg tablets**.

For descriptive purposes, we calculated competitor price changes by comparing their average price for the last quarter observed in the study to their average price in the quarter preceding the first RPI price observed. Competitor final price changes are presented as *percent price changes *(Figure 2, Table 2) and calculated as follows: ((average price_last quarter _- average price_quarter before RPI introduction_)/average price_quarter before RPI introduction_) × 100. We plotted examples of competitor price changes for medicines that exhibited price decreases, price increases, and no price changes (Figures 3, 4, 5, and 6). The Health Insurance Fund reimbursement prices are provided as a reference but are not meant to be an indicator of retail prices. The reimbursement price is the amount reimbursed to pharmacies by the Health Insurance Fund, whereby the patient pays the difference between the retail and reimbursement prices. Each medicine has a unique reimbursement price, ranging from 30-100% of the retail price. These reimbursement prices are changed regularly but these changes are typically not related to changes in retail prices.

**Table 2:** Descriptive results of RPI and competitor prices for the 30 highest volume insurance medicines before and after RPI introduction

Volume Rank*	Total # Claims (RPI and Competitor)		Generic Medicine Name (Brand Name)	Average Initial Price per Unit (SD) in Kyrgyz Som		Average Final Price per Unit (SD) in Kyrgyz Som		Competitor Price Change after RPI Introduction**
	**Before RPI Introduction**	**After RPI Introduction**		**Competitor**	**RPI**	**Competitor**	**RPI**	

1*	630	1,923	ampicillin 500 mg injection	7.6 (0.6)	6.0 (0.0)	7.7 (0.5)	6.0 (0.1)	+1.3%

2	0	494	amoxicillin 250 mg capsules	3.0*** (0.0)	2.5 (0.0)	3.0 (0.0)	2.5 (0.0)	0%

3*	467	1,138	benzylpenicillin 1 g injection	5.0 (0.0)	4.8 (0.2)	5.0 (0.0)	5.0 (0.2)	0%

4*	67	680	erythromycin 250 mg tablets	3.3 (0.2)	2.5 (0.0)	3.3 (0.4)	2.7 (0.0)	0%

5*	130	870	amoxicillin 250 mg tablets	3.0 (0.7)	2.6 (0.2)	2.8 (0.8)	2.5 (0.0)	-6.7%

6*	342	779	atenolol 50 mg tablets	1.6 (0.1)	1.1 (0.0)	0.9 (0.1)	0.9 (0.0)	-43.8%

7*	3	544	ciprofloxacin 250 mg tablets	4.3 (0.5)	2.4 (0.5)	3.5 (0.8)	2.8 (0.2)	-18.6%

8*	130	1,036	enalapril 20 mg tablets (Ednyt^®^)	10.5 (1.0)	5.4 (0.0)	8.1 (0.5)	8.1 (1.0)	-22.9%

9*	515	760	metronidazole 250 m tablets	1.0 (0.2)	0.8 (0.0)	0.9 (0.0)	0.6 (0.1)	-10%

10*	5	226	ferrous sulfate+folic acid+ascorbic acid tablets (Gyno-Tardyferon^®^)	3.8 (1.6)	8.2 (0.0)	8.2 (0.8)	8.2 (0.0)	+115.8%

11*	12	214	carbamazepine 200 mg tablets	1.9 (0.6)	2.0 (0.0)	1.5 (0.1)	1.4 (0.0)	-21.1%

12*	3	268	ferrous sulfate+ascorbic acid tablets (Taryferon^®^)	2.7 (0.0)	7.3 (0.0)	8.7 (1.2)	7.3 (0.0)	+222.2%

13	43	314	enalapril 10 mg tablets (Ednyt^®^)	6.9 (1.2)	5.1 (0.2)	5.0 (0.6)	5.2 (0.0)	-27.5%

14	150	429	co-trimoxazole 480 mg tablets	2.6 (0.3)	1.8 (0.0)	2.7 (0.3)	1.9 (0.0)	+3.8%

15	0	187	iron combination tablets (Ferum Lek^®^)	6.4** (1.0)	10.0 (0.0)	7.5 (1.9)	10.3 (1.3)	+17.2%

16*	53	209	nifedipine 20 mg retard tablets (Corinafar Retard^®^)	3.3 (0.9)	3.6 (0.3)	3.7 (0.7)	5.0 (0.0)	+12.1%

17*	7	49	Prednisolone 5 mg tablets	1.1 (0.1)	1.3 (0.4)	0.5 (0.0)	0.9 (0.5)	-54.5%

18*	138	193	diclofenac 25 mg tablets (Ortophen^®^)	0.7 (0.2)	0.4 (0.0)	0.6 (0.1)	0.6 (0.1)	-14.3%

19	114	197	ampicillin 250 mg tablets	1.9 (0.1)	1.4 (0.0)	1.5 (0.0)	1.6 (0.0)	-21.1%

20	484	395	co-trimoxazole 480 mg Tablets (Biseptol^®^)	4.0 (0.0)	2.2 (0.0)	3.5 (0.0)	3.8 (0.0)	-12.50%

21	43	74	drotaverine 40 mg tablets (No-Spa^®^)	2.4 (0.2)	2.3 (0.0)	2.5 (0.0)	2.6 (0.0)	+4.2%

22	28	110	metronidazole 250 mg tablets (Trichopol^®^)	3.5 (0.4)	2.1 (0.0)	3.5 (0.0)	3.5 (0.0)	0%

23*	73	548	metronidazole 500 mg vaginal suppositories	11.9 (5.3)	5.2 (0.0)	6.5 (0.8)	6.0 (0.0)	-45.4%

24	13	60	ferrous sulfate+ascorbic acid drag (Ferroplek^®^)	1.5 (0.5)	0.8 (0.0)	1.5 (0.0)	1.6 (0.0)	0%

25*	445	927	diclofenac 75 mg injection	8.1 (1.0)	3.9 (0.4)	6.0 (0.2)	5.3 (0.3)	-25.9%

26	140	226	bromhexine 8 mg tablets	0.5 (0.1)	0.3 (0.0)	0.4 (0.1)	0.6 (0.3)	-20%

27*	64	336	omeprazole 20 mg capsules	5.9 (0.5)	5.1 (0.6)	3.4 (1.0)	2.7 (0.0)	-42.4%

28*	70	113	ketotifen tablets	1.4 (0.3)	0.8 (0.0)	0.8 (0.0)	0.8 (0.0)	-42.9%

29*	269	216	doxycycline 100 mg capsules	2.8 (0.0)	1.3 (0.2)	1.0 (0.0)	1.5 (0.0)	-64.3%

30	3	56	verapamil 80 mg tablets	2.2 (0.5)	1.2 (0.0)	1.6 (0.0)	1.7 (0.0)	-27.3%

*Used in time-series analyses

**Difference in competitors' price from the quarter before the RPI was introduced to the end of the study period

***No price for quarter before RPI intro; this price from quarter where RPI price first appears

We conducted multiple regression analysis on 19 of the top 30 selling medicines which met our sample size inclusion criteria that required at least seventeen quarters of competitor price data, including three quarters of data before RPI introduction and at least nine quarters of RPI price data. In quarters with missing data due to sparse purchases, we imputed the price and number of transactions using the adjacent quarters.

We estimated competitor prices for 3 time periods: the immediate price change from the quarter before the RPI was introduced to the quarter after the RPI was introduced (Table 3, Column B), quarterly trends prior to the RPI introduction (Table 3, Column C), and the long term quarterly price trends after the RPI introduction (Table 3, Column D). When quarterly price trends before RPI introduction are equal to quarterly price trends after RPI introduction, we present the overall quarterly rate of change (Table 3, Column E). Statistical significance is defined as p ≤ 0.05.

**Table 3:** Multivariate results of competitor price trends for 19 medicines before and after RPI introduction

		Price Trends Before RPI are NOT Equal to Price Trends After RPI		Price Trends Before RPI are Equal to Price Trends After RPI
**A**	**B**	**C**	**D**	**E**
**Medicine**	**Immediate price effect of RPI**^**§**^	**Quarterly price trends before RPI**	**Quarterly price trends after RPI**	**Quarterly price trends before and after RPI**

ampicillin 500 mg injection	-1.19**	-0.27**	0.01	

atenolol 50 mg tablets	-0.52**	-0.14*	-0.05	

metronidazole 250 mg tablets	-0.35**	-0.03**	0.01	

carbamazepine 200 mg tablets	-1.04*	-0.22*	-0.02	

diclofenac 75 mg injection	-2.33**	-0.34**	-0.07	

doxycycline 100 mg capsules	-1.19**	-0.08**	-0.03	

benzylpenicillin 1 g injection	0.06*			-0.02**

erythromycin 250 mg tablets	-0.07			-0.01

amoxicillin 250 mg tablets	0.20*			-0.05**

ciprofloxacin 250 mg tablets	-0.73*			-0.01

enalapril 20 mg tablets (Ednyt^®^)	-0.18			-0.21**

ferrous sulfate+folic acid+ascorbic acid tablets (Gyno-Tardyferon^®^)	1.96*			0.26**

ferrous sulfate+ascorbic acid tablets (Taryferon^®^)	1.40			0.29**

nifedipine 20 mg retard tablets (Corinafar Retard^®^)	0.93*			-0.06

prednisolone 5 mg tablets	0.38			-0.10*

diclofenac 25 mg tablets (Ortophen^®^)	-0.05			-0.01*

metronidazole 500 mg vaginal suppositories	-4.85**			-0.27**

omeprazole 20mg capsules	0.75*			-0.30**

ketotifen tablets	0.18			-0.08**

^§ ^Difference in competitor price from the quarter before to the quarter after the RPI was introduced

*p ≤ 0.05

**p < 0.0001

We conducted model checking diagnostics, including a test for residual autocorrelation, to ensure our model was appropriate for our distribution. Our model takes into account price dispersion because it utilizes all price values, not just average price. But we present the average price to facilitate interpretation of the results. We conducted all descriptive and multivariate analyses using SAS 9.1 (SAS Institute, Carey, NC).

## Results

Descriptive analyses reveal that prices for 21 of 30 (70%) competitor medicines changed by at least 10% to mimic RPI prices after the introduction of the RPI. Prices for 17 of 30 (57%) competitor medicines decreased at least 10%, with price decreases ranging 10-64.3% (Figure 2). Prices for 4 of 30 (13%) competitor medicines increased more than 10%, with price increases ranging 12.1-222.2%, apparently in response to RPI prices introduced at higher rates than those charged by competitors. Nine of 30 (30%) competitor medicines revealed price changes +/- 9.9% after the RPI introduction.

Detailed information on specific medicine price changes is provided in Table 2. Among the seventeen competitor medicine prices that decreased at least 10% after RPI introduction, six, seven, and four medicines showed price reductions of 41-64%, 20-40%, and 10-19%, respectively. Among the four competitor medicines that increased more than 10% after RPI introduction, three were iron-containing medicines.

Two examples where competitor medicine prices decreased after RPI introduction are provided in Figures 3 and 4. Figure 3 reveals dramatic competitor price reductions for metronidazole 500mg vaginal suppositories where the competitor price decreases immediately from 11.9 Kyrgyz Som/suppository prior to RPI introduction to 7.9 in the following quarter. The quarterly trends continue downward to a final price of 6.5 Kyrgyz Som/suppository at the end of the study period.

In Figure 4, the competitor price for enalapril 20mg tablets (Ednyt^®^) was 10.5 Kyrgyz Som/tablet in the quarter prior to the introduction of RPI pharmacies. The RPI entry price was 5.4 Kyrgyz Som but they soon increased their price to 8.1 Kyrgyz Som. A few quarters later, the competitors priced their product to mimic the RPI prices, also ending at 8.1 Kyrgyz Som at the end of the study.

Figure 5 illustrates dramatic price increases observed for tablets containing iron and ascorbic acid (Gyno-Tardyferon^®^) in response to these products being sold at higher prices in RPI pharmacies. Competitor prices increased from 3.8 Kyrgyz Som/tablet to 8.2 Kyrgyz Som/tablet after the RPI pharmacies introduced the product at the price of 8.2 Kyrgyz Som/tablet.

Lastly, figure 6 provides an example of a medicine that exhibits no overall price change from the quarter before the RPI is introduced and the end of the study period. While competitor prices fall for a brief period of time, the pharmacies eventually revert to prices charged prior to the introduction of the RPI.

Multivariate analysis revealed fourteen of nineteen (74%) competitor medicines with significant price changes from the quarter before RPI introduction to the quarter after RPI introduction (Table 3, Column B). Nine of the nineteen (47%) medicines revealed price decreases, which ranged from ranged from 0.35-4.85 Kyrgyz Som per unit, while five of the nineteen (26%) revealed price increases, ranging from 0.06-1.96 Kyrgyz Som per unit.

Interestingly, among medicines with differences in price trends before and after the RPI introduction, 6 of 6 (100%) revealed downward price trends before the RPI (Table 3, Column C). All six of these medicines revealed significant price decreases immediately after RPI introduction (Table 3, Column B) with long term prices remaining relatively unchanged (Table 3, Column D).

Mixed results are noted among thirteen medicines with no differences in price trends before and after RPI introduction. Seven of these 13 (54%) medicines showed downward price trends, while prices for 3 of the 13 (23%) trended upward (Table 3, Column E). For most of these medicines, the changes in price trends over time (Table 3, Column E) are far less than those observed immediately after the introduction of the RPI (Table 3, Column B).

## Discussion

This study confirms the success of the RPI as an innovative, not-for-profit option for promoting medicine price competition in Kyrgyzstan, and ultimately increasing access to medicines. The RPI not only addressed geographic access by enabling rural residents to buy medicines in their own villages, it also spurred dramatic price competition in private pharmacies located in the district center. Thus, the RPI's impact was far greater than anticipated as the new pharmacies managed also to increase access to medicines in other villages through the competitive price reductions they engendered. The ultimate result was more affordable medicine for both villagers and residents of the district center. It is worth noting, however, that for some medicines, when the RPI introduced prices higher than competitor prices, the competitor increased their prices to match those of the RPI. In this regard, the RPI demonstrates power to drive markets both downward and upward in price.

We do not believe the RPI resulted in the institution of a rural market. Instead, we believe the market already existed in Jumgal prior to the RPI and that the entry of the not-for-profit RPI spurred a more competitive market. Indeed, demand for medicines was already documented before the RPI when villagers identified access to medicines as their number one health concern. On the supply side, medicines were available in the rayon center, but not in the villages themselves. Prior to the establishment of the RPI, villagers either hired a taxi to deliver medicines from existing pharmacies to their homes or they secured some means of transport to travel outside the village to the nearest pharmacy. The establishment of the RPI, therefore, took business away from the existing pharmacies despite being located far away. The emergence of the RPI pharmacies also seems to have stimulated expansion of the private sector into new villages. Owners of pharmacies in the rayon center opened two branch pharmacies in two of the larger villages, perhaps in an attempt to minimize loss of business in the rayon center.

While we have no means to assess price collusion, we suspect that some degree of collusion existed among the private pharmacies that were established prior to the RPI. We therefore suspect the introduction of the RPI disrupted any existing price collusion in the region. Given that our study tracks prices for three years after the establishment of the RPI, we suspect the competitors' price reactions are sustained and not a one-off reaction to the RPI.

When the competitors change prices to mimic RPI medicine prices, their prices are often near identical to the RPI prices. We were unable to fully ascertain how the competitors gained market intelligence on RPI medicine prices. Upon establishment of the RPIs, the RPI nurses were instructed to display all medicines with price tags affixed to their packages. When interviewed a few months later, the nurses told investigators that they no longer displayed medicine prices because employees from competitor pharmacies would visit RPI pharmacies and record medicine prices. The RPI nurses, management, and others were greatly concerned about predatory pricing by competitor pharmacies. The RPI was quite fragile when first established and many feared the existing private sector pharmacies would intentionally undercut RPI prices in order to drive them out of business. Our results suggest that competitors are still obtaining price information from RPI pharmacies even though price tags are no longer affixed.

While our study provides compelling results, it has limitations. First, we were only able to access prices for those medicines covered by the Health Insurance Fund. The sale of all medicines covered by the Health Insurance Fund, however, accounted for 51% of total RPI revenue in the fourth quarter of 2007 [41]. Because these pharmacies serve sparsely-populated rural regions, small sample sizes limited us to analyzing only 19 of the 30 top-selling medicines with multivariate methods. Some medicines that passed our sample size criteria for multivariate analysis had limited insurance claims prior to or after RPI introduction. We utilized a linear model that assumes a relatively constant quarter-on-quarter price change. Given that we only observed seventeen quarters of data, we believe the linear assumption is reasonable. Model checking diagnostics, such as test for residual autocorrelation, showed that our model is adequate for our purposes.

Furthermore, we note that to make optimum use of space, we provided price trends in graphic form for only four of the thirty medicines studied, however a full set of figures is provided in Additional File 1. Figures provided in this paper depict the three types of competitor price changes observed after the introduction of the RPI-price decreases, price increases, and no price changes-and are representative of the medicines in each price change category. We had no means to determine why some medicines exhibited dramatic price changes and others remained unchanged. While we had no data on the quality of medicines, we do not believe that quality confounded our price findings. For branded generic medicines, we assume the quality of medicines to be identical in RPI and non-RPI pharmacies. Because the RPI and non-RPI pharmacies purchase medicines from the same wholesalers in Bishkek, we assume quality of non-branded generic medicines is comparable. We have no evidence of pharmacies over-charging insurers, however we expect it happens to some degree. Over-charging is less likely to happen within the RPI because it is supervised by staff from the Mandatory Health Insurance Fund.

While research on market impact is typically complicated due to many concurrent interventions and changing market conditions, we are confident that the market in Jumgal is truly local and has no other large-scale interventions that might be responsible for our findings. Indeed, even an annual inflation of 10% [42] did not seem to affect medicine prices in this region over the study period. We decided to use current medicine prices in lieu of adjusting prices for inflation after noting that most medicine prices trended downward or remained unchanged over the 4 years and did not seem to follow the national inflation rate. We are not sure why medicine price trends are inconsistent with national inflation trends. We suspect that medicine prices were already priced at the highest prices the market could bear or that national inflation rates simply do not represent the price trends in rural medicine markets. Lastly, we have no means of determining if the market existing prior to the RPI was competitive or collusive with regards to price setting.

Our study was designed only to assess whether the RPI induced regional price competition and not to evaluate rational use of medicines in RPI pharmacies. Others have shown that perverse incentives to misuse medicines may result when prescribers benefit from medicine sales [43]. In this study, we note that ampicillin injection has the highest sales volume and the largest number of insurance claims (Table 2), suggesting overuse of both antibiotics and injections. Additional research is needed to assess the impact of the RPI on rational use of medicines.

Similarly, our study does not aim to explain why the RPI has been successful or how the RPI has been sustainable amidst other documented failures to increase access to medicines in rural regions. The RPI may offer a model that can be scaled up across many more regions and in other parts of Central Asia, but it is important to first determine the most critical elements that led to its success. While we cannot pinpoint theses critical components, we note the key characteristics of the RPI, including the role of highly trained and motivated players who genuinely wanted to develop and test new ways to increase access to essential medicines in rural areas. These staff devoted a great deal of their personal time and energy to seeing the project through, and it would be a mistake to overlook or underestimate the value of this social capital, especially in post-Soviet Central Asia. From a social and political standpoint, the climate of Kyrgyzstan is recognized as being more conducive to civil society than that of any of its neighbors in Central Asia. However, with the exception of a few very strong professional organizations, the country's health sector NGOs tend to be less evolved than the NGO managing the RPI. In addition, the NGOs tend to be rather fractured, often working from outside the government rather than in collaboration with it. We therefore see a need for donors, international organizations, and governments to assist in reorganizing and building the capacity of existing NGOs to refocus their social capital toward more concrete activities, such as establishing and overseeing the RPIs.

Understanding that the majority of people in developing countries still seek care from pharmacies rather than public sector health facilities, many donors have been eager to develop private sector interventions but have been wary of engaging directly with the private sector. At the same time, donors are eager to establish and promote community-based programs and civil society. The non-profit nature and involvement of civil society organizations in the rural pharmacy initiative model could provide donors with an opportunity to accomplish multiple goals without compromising their non-profit missions.

Lastly, the study reveals the utility of data on medicines that are routinely collected through mechanisms such as insurance schemes. These types of data sources are rich and should be used to build a solid body of evidence to guide policy on access to medicines for the poor. Research on interventions to increases access to medicines must include assessment of potential impact on both formal and informal markets. More work is needed to identify incentives for NGOs and other non-profits to engage in the establishment and management of rural pharmacies that can compete with existing private pharmacies. This should include determination of the operating costs to establish and maintain rural pharmacies and the minimum mark-ups needed to sustain these pharmacies, as well as pricing policies that promote rational use of medicines. Additional research is also needed to examine policies and programs that promote and impede competition in the pharmaceutical sector, including description of market size and structure, presence or absence of competition laws, price regulation, barriers to market entry, and marketing.

## Conclusion

Initiatives designed to increase equitable access to medicines in rural regions of developing and transitional countries should consider the potential to leverage medicine price competition as a means of achieving their goal. The inclusion of civil-society organizations and non-governmental organizations in the design and management of these initiatives, in collaboration with governments and international organizations, provides opportunities for capacity building, health sector development, and business development in rural regions that are often neglected.

## Competing interests

The authors declare they have no competing interests.

## Authors' contributions

BW designed and coordinated the study, participated in data cleaning and data analysis, and was the lead author on this paper. JM coordinated data collection and management, and participated in preparation of the manuscript. MG conducted data cleaning and data analysis and participated in preparation of the manuscript. YT assisted in data analysis and participated in preparation of the manuscript. RL and HL participated in the preparation of the manuscript. All authors read and approved the final manuscript.

## Supplementary Material

Additional file 1 **Price trends for thirty top-selling medicines**. RPI and non-RPI average price comparisons for the thirty top-selling medicines Click here for file
