# Supplementary material for: Towards equitable access to medicines for the rural poor: analyses of insurance claims reveal rural pharmacy initiative triggers price competition in Kyrgyzstan
Source: Int J Equity Health. 2009 Dec 14;8:43. doi: 10.1186/1475-9276-8-43 (PMC2803474; doi:10.1186/1475-9276-8-43)
Supplement: Supplementary file 1 [file 1475-9276-8-43-S1.DOC]

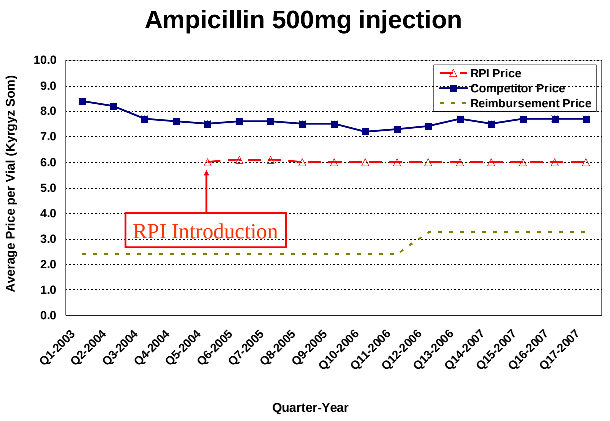
Ampicillin 500mg injection
Average Price per Vial (Kyrgyz Som)
RPI Price
Competitor Price
Reimbursement Price
RPI Introduction
Quarter-Year

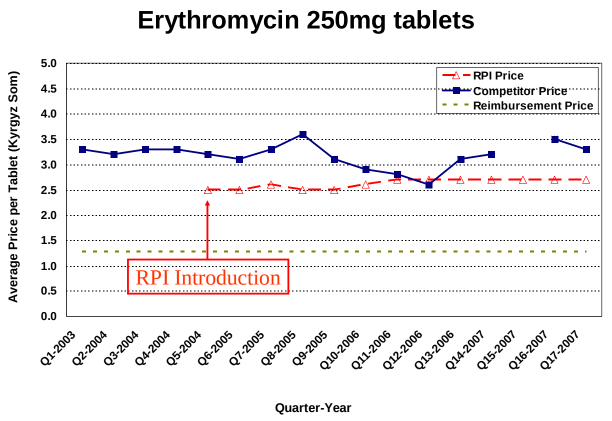
Erythromycin 250mg tablets
Average Price per Tablet (Kyrgyz Som)
RPI Price
Competitor Price
Reimbursement Price
RPI Introduction
Quarter-Year

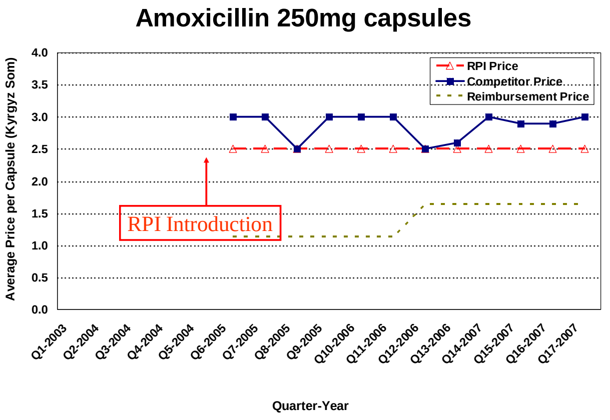
Amoxicillin 250mg capsules
Average Price per Capsule (Kyrgyz Som)
RPI Price
Competitor Price
Reimbursement Price
RPI Introduction
Quarter-Year

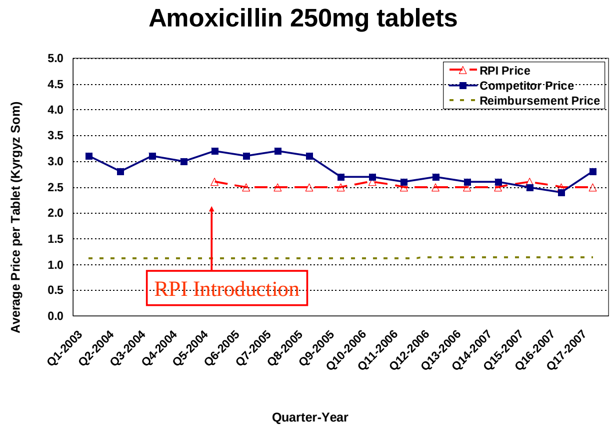
Amoxicillin 250mg tablets
Average Price per Tablet (Kyrgyz Som)
RPI Price
Competitor Price
Reimbursement Price
RPI Introduction
Quarter-Year

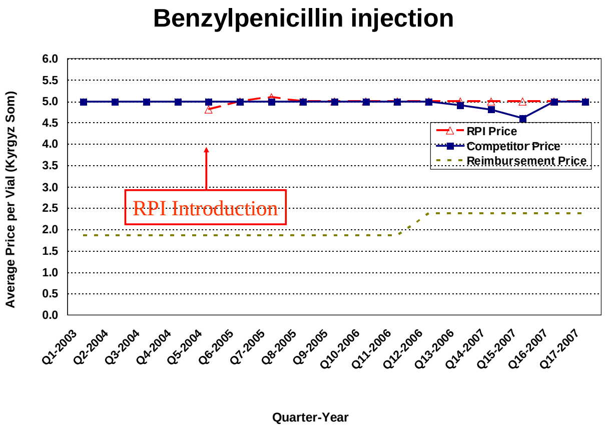
Benzylpenicillin injection
Average Price per Vial (Kyrgyz Som)
RPI Price
Competitor Price
Reimbursement Price
RPI Introduction
Quarter-Year

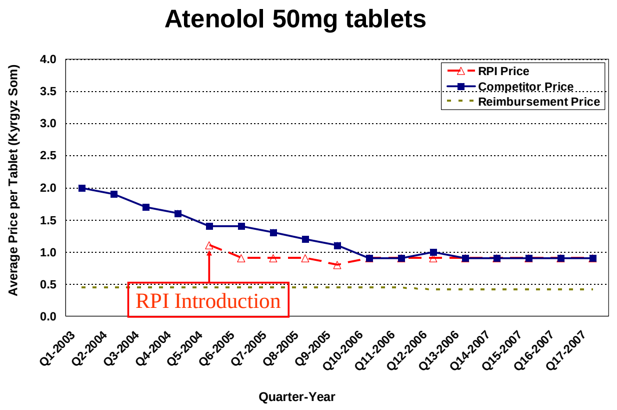
Atenolol 50mg tablets
Average Price per Tablet (Kyrgyz Som)
RPI Price
Competitor Price
Reimbursement Price
RPI Introduction
Quarter-Year

Ciprofloxacin 250mg tablets

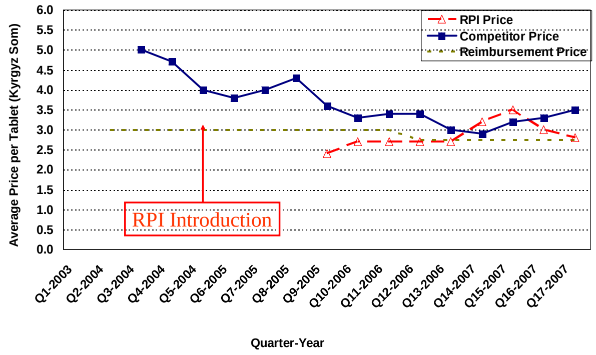

Ferrous Sulfate+ Gyno-Tardyferonum® 80 tablets

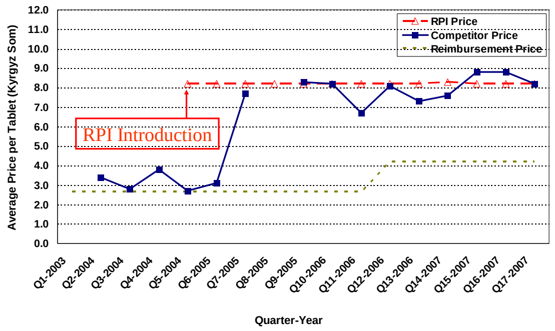

Enalapril 20mg tablets (Ednyt®)

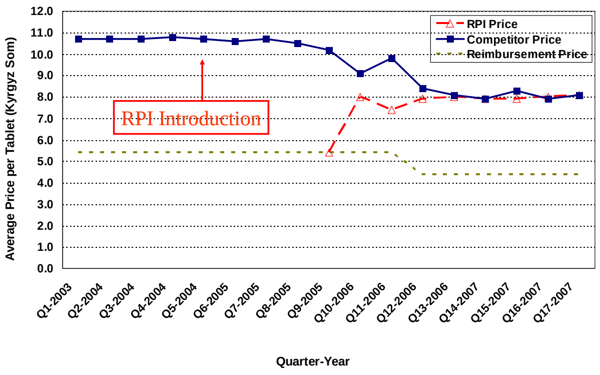

Carbamazepine 200mg tablets

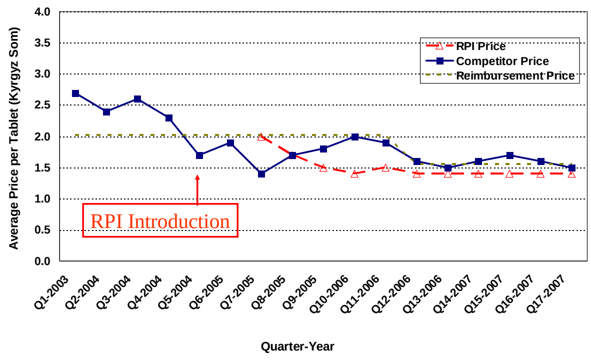

Metronidazole 250mg tablets

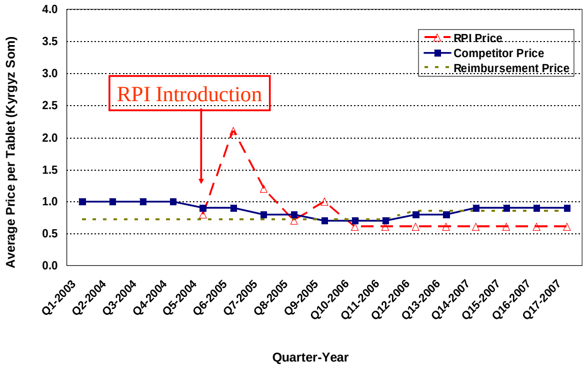

Tardyferonum® 80 tablets

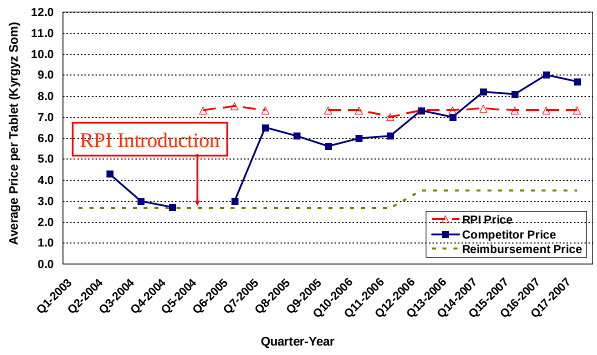

Enalapril 10mg tablets (Ednyt®)

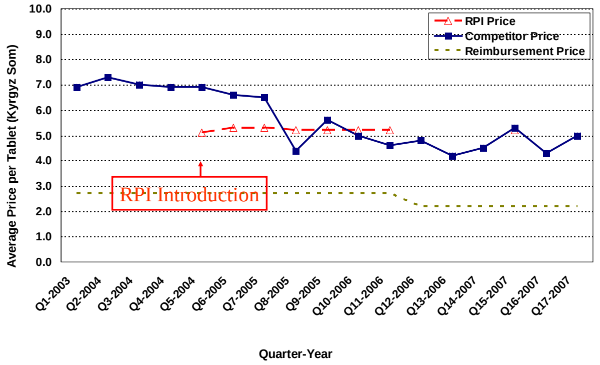

Nifedipine Long Acting 20mg tablets (Corinfarum Retard®)

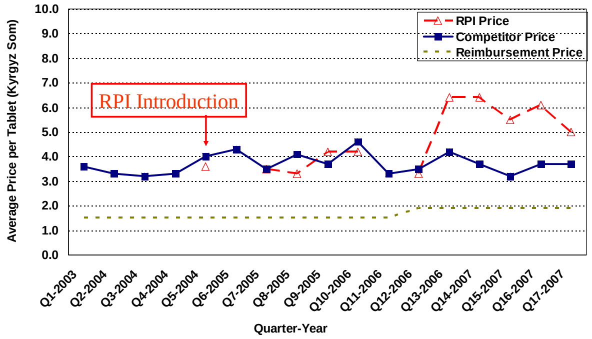

Co-trimoxazole 480 tablets

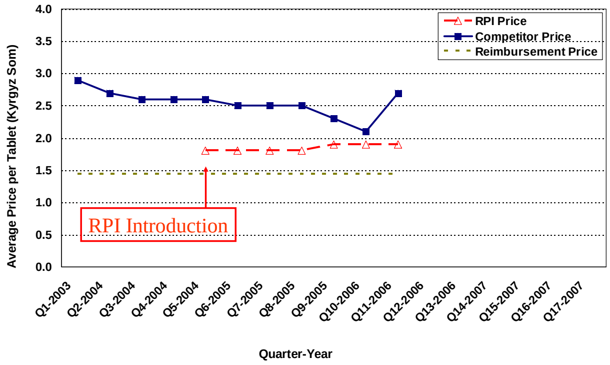

Prednisolone 5mg tablets

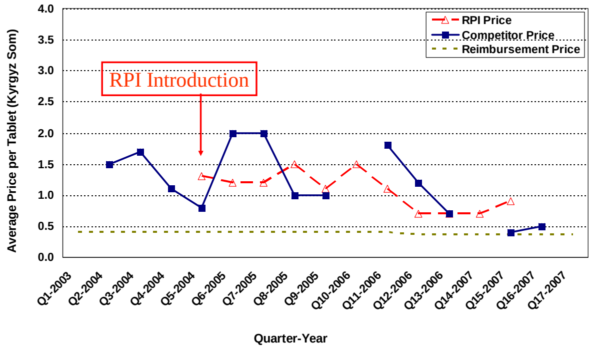

Ferrum Lek 100 tablets

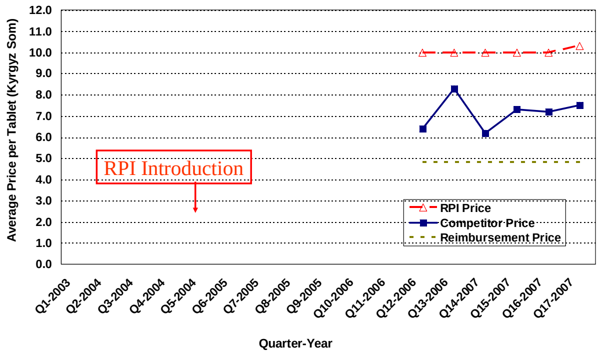

Diclofenac 25mg tablets (Ortophenum®)

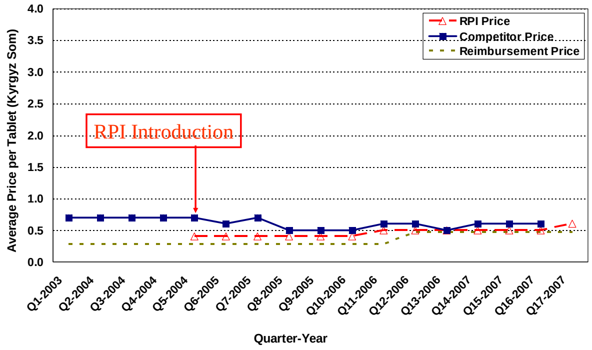

Ampicillin 250mg tablets

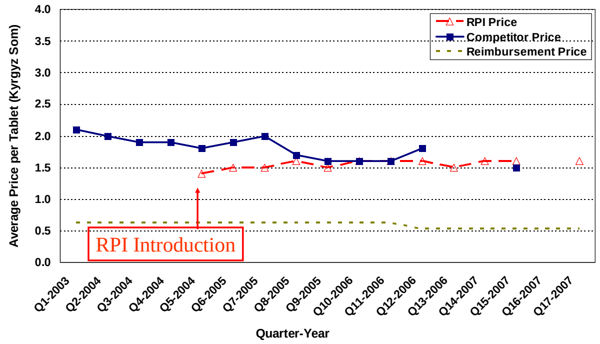

Metronidazole 250mg tablets (Trichopolum®)

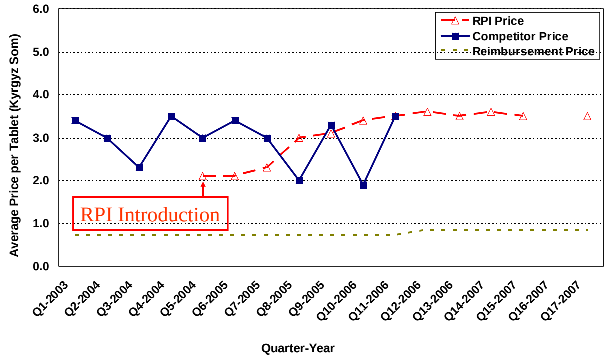

Cotrimoxazole 480 tablets (Biseptol®)

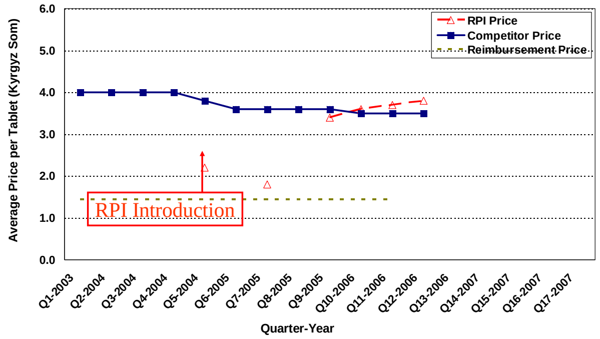

Metronidazole 250mg tablets (Trichopolum®)

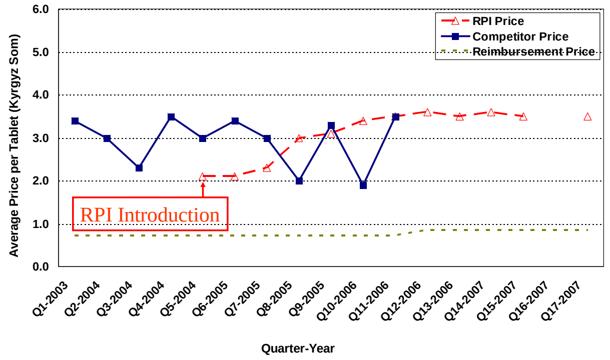

Drotaverine 40mg tablets (No Spa®)

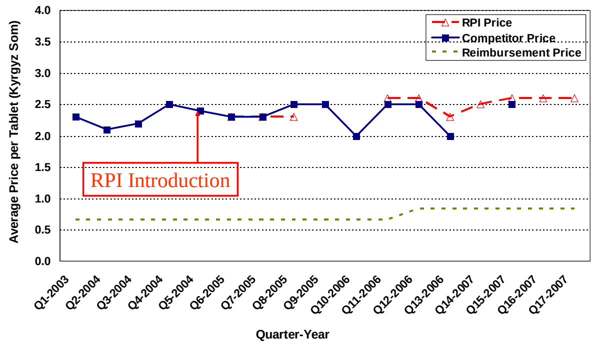

Ferroplektum 10 drag

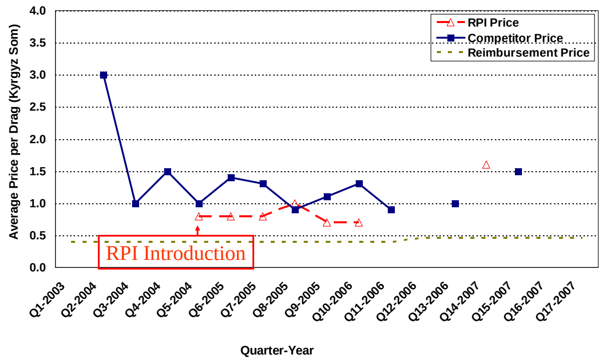

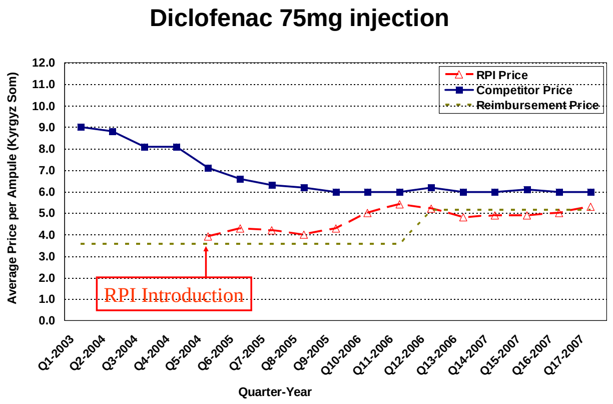
Diclofenac 75mg injection
Average Price per Ampule (Kyrgyz Som)
Quarter-Year
RPI Price
Competitor Price
Reimbursement Price
RPI Introduction

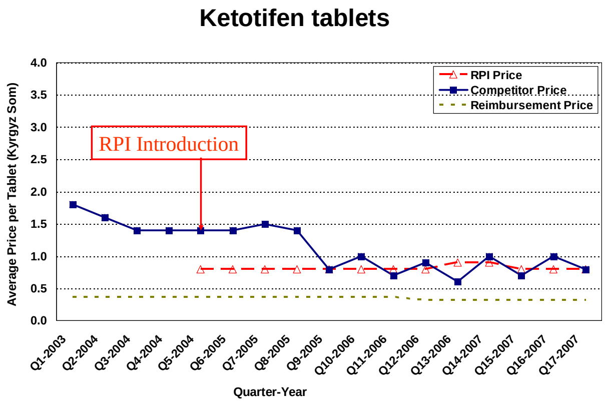
Ketotifen tablets
Average Price per Tablet (Kyrgyz Som)
Quarter-Year
RPI Price
Competitor Price
Reimbursement Price
RPI Introduction

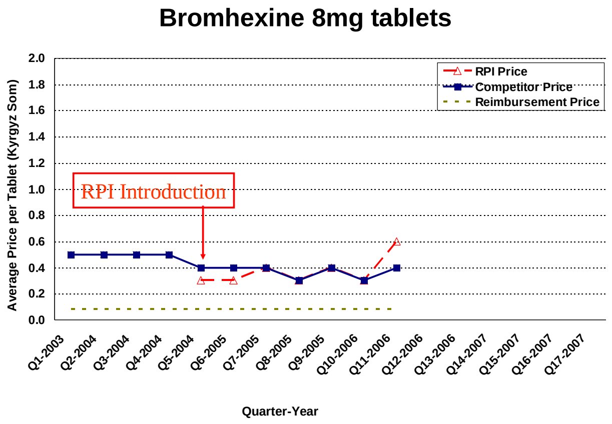
Bromhexine 8mg tablets
Average Price per Tablet (Kyrgyz Som)
Quarter-Year
RPI Price
Competitor Price
Reimbursement Price
RPI Introduction

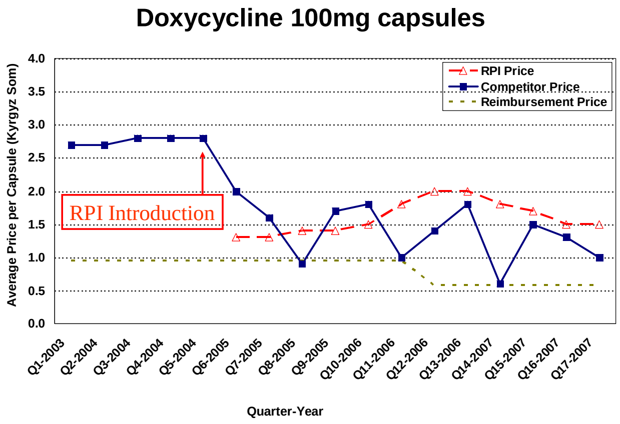
Doxycycline 100mg capsules
Average Price per Capsule (Kyrgyz Som)
Quarter-Year
RPI Price
Competitor Price
Reimbursement Price
RPI Introduction

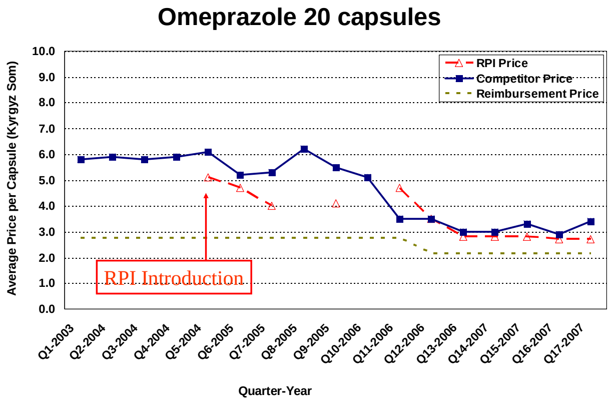
Omeprazole 20 capsules
Average Price per Capsule (Kyrgyz Som)
Quarter-Year
RPI Price
Competitor Price
Reimbursement Price
RPI Introduction

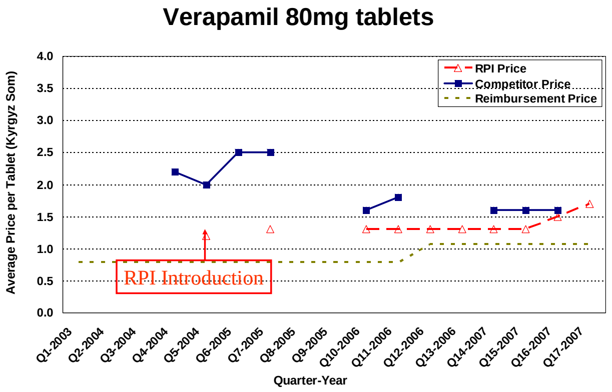
Verapamil 80mg tablets
Average Price per Tablet (Kyrgyz Som)
Quarter-Year
RPI Price
Competitor Price
Reimbursement Price
RPI Introduction
